# Address of the President of the International Hahnemannian Association, at Its Annual Meeting at Niagara Falls, June 27th, 1899

**Published:** 1899-07

**Authors:** Walter M. James


### HOMŒOPATHIC PHYSICIAN,

A MONTHLY JOURNAL OF

HOMŒOPATHIC MATERIA MEDICA AND CLINICAL MEDICINE.

"If our school ever gives up the strict inductive method of Hahnemann, we are lost, and deserve only to be mentioned as a caricature in the history of medicine."—CONSTANTINE HERING.

Vol. XIX. JULY, 1899. No. 7.

### ADDRESS OF THE PRESIDENT OF THE INTERNATIONAL HAHNEMANNIAN ASSOCIATION, AT ITS ANNUAL MEETING AT NIAGARA FALLS, JUNE 27th, 1899.

FELLOW-MEMBERS: The present occasion finds us once more assembled to do honor to the cause which we espouse, and to compare notes and contribute to each other's knowledge in the new and great method of healing the sick which we call Homœopathy.

Our beloved system has instructed us, not alone in the best way of selecting remedies to relieve immediately the suffering which we see about us and to terminate favorably the diseased states from which that suffering arises, but also enables us to take more just and more comprehensive views of the phenomena of drug action, the scope and meaning of the action of poison, the just importance of that new fad in medicine, auto-intoxication, and the real truth of the famous germ theory.

Last and greatest of all it enables us to form clear conceptions of the nature and origin of disease in the human system.

A man standing close to the foundations of a great edifice is not in a position to comprehend the plan of its building, its proportions, or the beauty of its design. He must get away from it to a convenient distance that will enable him to include its height and its breadth in one angle of vision, before the true idea of its character can enter his mind.

The traveler toiling along some lonely trail that leads him from one cañon to another, from a pass in the mountains to some river valley, where his path winds in and out through tangled bushes, "beds of reeds, and many a fen where the serpent feeds," later into some darkened forest glade, and thence over hills and mountains, in endless confusion, can form no idea of the character of the country, the trend of landscape, the direction of the streams, the relationship of the mountains, or the extent and luxuriance of the vegetation. He must get above his surroundings; he must climb some mountain peak, or attain some great plateau, which will enable his eye to find a wider horizon before the full panorama of the country bursts upon his vision and gives rise to the comprehensive idea which will enable him clearly to understand the scene about him.

Applying these images to the question of the nature of disease and the relationship of remedial measures to it, it can be said that the man who slowly and painfully studies the phenomena of one form of disease or who spends all his time in gazing down the tube of a microscope at some pathological growth; or who concentrates his whole attention upon the growth, development, and variation of the cell, to the exclusion of the whole phenomena of human life, human history, human sufferings, and the wonderful spectacle of man's occult characteristics is like the spectator who stands in the shadow of the palace, or the wayfarer in the valley, oblivious —the one to the grandeur of the building, the other to the beauty and diversity of the landscape.

It is not intended in these remarks to detract from and discourage the numerous minute investigations that are being pursued in various channels and by various means; notably the microscope, the spectroscope, and the Roentgen rays, to enlarge the boundaries of human knowledge and assist in the problem of curing the sick.

All that it is sought here to convey to the mind is that these workers in special fields of scientific research are not in a sufficiently lofty position, have not a sufficiently wide intellectual horizon to enable them to form a generalization that is at once logical and in consonance with the facts.

All who deny heredity in disease are open to the force of these criticisms.

All who seek in the phenomena of perverted cell action an explanation of the singular phases of sickness, all who persistently refuse to leave the material plane in studying these actions are alike vulnerable to such criticisms.

Hahnemann by his remarkable comprehensive philosophy, by his keen analysis, his patient research, his rare historic knowledge, was enabled to form a brilliant generalization, that, in its results, is like taking us on to a high mountain-top where we may view the landscape and get a map of the devious road we have traversed.

By his scientific generalization we are enabled to bring about an orderly arrangement in our own minds of the many different phenomena and isolated occurrences that without his instruction would be impossible to comprehend.

These thoughts are suggested by the news received in this country that the illustrious Professor Virchow, the father of cellular pathology, in an address before the Tuberculosis Congress, declared there was no evidence that tuberculosis was inherited, as he had never in the course of his microscopical investigations found any trace of its presence in the unborn babe, though he admitted it could acquire the disease in one day after birth.

The information referred to is contained in a cable dispatch to the *Evening Telegraph* of Philadelphia, Pa., and was published in that journal on Saturday evening, May 27th, 1899. The dispatch is as follows:

"BERLIN, May 27th.—Professor Leyden gave a brilliant reception last evening to the delegates to the Tuberculosis Congress, which was attended by the Imperial Chancellor, Prince von Hohenlohe, the foreign diplomatists, and other notable persons.

"Yesterday afternoon Professor Rudolph Virchow delivered an important address before the Congress, rejecting the theory of hereditary tuberculosis. This doctrine, he declared, was contradicted by all his pathological researches. He said he had never found tuberculosis in unborn or newborn infants, though it might be contracted during the first day's existence."

According to this celebrated pathologist there can be no possibility of future tuberculosis, no potentiality of tuberculosis, as electricians would say, unless there be traces of the condition under the microscope in the fœtus. His conclusion is thus diametrically opposite to general opinion, to the observed phenomena in the experience of many of us and to the revelations made by Hahnemann's own observations and subsequent generalizations.

Though I am not an investigator, nor in late years have I worked at all with the microscope, yet I venture to disagree with Professor Virchow in his conclusions, and consider them based on insufficient evidence. Because there is no visual trace of tuberculosis in the fœtus, that is no guarantee of the absence of tendency to tuberculosis. The *potentiality* of energy that would culminate in tuberculosis can reside in the cell life of the fœtus without our being able to perceive it.

Potentiality of energy without visibility frequently happens in the scientific world.

When the two poles of a galvanic battery are kept separated, they are apparently without any power whatever. Bring them into contact with each other and action is at once apparent. A spark passes, magnetism develops, electrolytic decomposition occurs, and other phenomena. It would be folly to deny this power lying latent when the poles are separated merely because not seen.

It is possible to so impel a billiard ball along a billiard table by suitable impulses from the hand, without the aid of a cue, that the ball will travel two-thirds of the length of the table, then stop, seem to reflect, and return upon its course.

Now when that ball starts on its journey forward it appears to have only one impulse: that a motion which would seem to carry it the whole length of the table. Yet concealed within its mass is a second impulse which will, at the proper moment, assert itself, overmaster the forward motion, destroy the latter, and finally exert its own power to cause the return of the ball to the starting point.

A spectator might be supposed to be skeptical concerning the presence of this second impulse within the ball, and seeing no manifestation of it during the forward motion of the ball, might deny its existence and might predict that the ball would fail to return to its starting point.

In the same way a bowler at base-ball can give the ball a combination of two or more impulses, in obedience to one of which it will proceed directly toward the batsman, but having arrived in that neighborhood will suddenly deflect and avoid the bat, going round it.

The spectator unfamiliar with these possibilities in pitching a ball might be supposed to be skeptical of the existence of these impulses, might have time to deny their existence, and argue against the possibility of such a result. Such we think is the attitude of Professor Virchow.

Now the human being is projected into this world by inscrutable natural forces, under conditions which may be symbolized by the billiard ball or the base-ball projected from the hand. The human projectile has an initial impulse which will send it along the path of life through childhood, manhood, and old age. Superposed upon this impulse is a secondary influence which will stop its career at a certain point and cause its return toward that mysterious oblivion from which it came. With each individual who thus comes into the world the secondary impulse is greater or less, taking him a greater or less distance. In one this secondary impulse is represented by cancer. In another it is tuberculosis, in another, it is a tendency to meet with accident, and this last being noticed and recognized is called a "fatality." Whatever the form in which it come to the individual it is of the nature of a secondary impulse that tends to limit his progress through the world and cause his return to the infinite. These secondary impulses are exceedingly numerous and were comprehensively included by Hahnemann under three great classes, Psora, Syphilis, and Sycosis.

Here then we get a new view of the greatness of Hahnemann's mind, the depths of its workings, and the beauty of his generalizations.

Tuberculosis belongs to the class of psora. Its impress upon an individual human germ before birth is an invisible potentiality which according to the force of its original impulse will declare itself in one case in youth, in another in adult life, and in still another in old age.

Any scientific observer who declares its non-existence because of the absence of obvious indications places himself in the attitude of the bystander who being told of the several impulses within the mass of the billiard ball or the base-ball, denies the existence of all except the initial impulse, because he cannot see them.

This is the position in which the learned Professor Virchow has placed himself to-day. Yet in spite of his denial these secondary impulses are acting right under his eyes and are cutting off myriads, and he himself displays his misgiving of the truth of his assertion by the saving clause that "it (tuberculosis) might be contracted during the first day's existence."

The subject does not end here.

If, as Professor Virchow says, there is no evidence that tuberculosis is inherited, adopting his view then the other explanation must be correct—that it is a germ and that it is therefore contagious. If so, how does it happen that one person should be affected by it and another one should escape? Why are not all the members of a family attacked with tuberculosis when one is obviously suffering from it?

There is undoubtedly a selective affinity exercised to bring about the discrepancies which we perceive on every hand. What is the origin of that selective affinity?

Plants generally need a suitable soil for their successful growth. Germs or bacteria or bacilli *are* plants, and must therefore have suitable soil. If tuberculosis be a germ it is not different in needing a suitable soil. The human system is gifted with remarkable resistance to the incursions of all sorts of poison, parasites, and injuries. Why is it not universally and invariably so? The answer lies in the fact that there are influences which have entered into and deteriorated this resisting power. These influences are inherited.

This answer to our question brings us back to the doctrine of the miasms of our own school of medicine. Hahnemann himself has furnished the answer in his conception of the three great inherited miasms, psora, syphilis, and sycosis. These are the influences which have broken down the resisting power of the system and so enabled germs to find a lodgment and perpetrate their ravages, as we see in the inroads made by tuberculosis.

Here we have a clear reason why tuberculosis should attack one person and neglect another; and no matter whether the view be adopted that it is an inheritance or that it is an infecting germ, it all resolves itself into the great fact that the supreme influence which determines its action is an inheritance of deteriorating tendencies which furnish a soil in which the poison or the germ finds suitable nourishment and encouragement for growth and propagation. In what way are these inheritances transmitted from parent to offspring?

Is it a perverted cell that generates its own kind, with always the same perversion; and which enters the fœtus whilst yet in the uterus; and that continues to propagate its own species after the birth of the matured child? Such is, evidently, the view held by Professor Virchow, his not finding this perverted cell leading him to a conclusion that is directly opposed to almost universal experience.

Is it *anything* material that is transmitted? I do not think so. The influence impressed upon the father or mother or both is what may be called a "tendency." It is an energy imparted to the cell life; an energy of a perverted character, that as soon as the offspring starts out on an independent life, and begins to assimilate food and to propagate true physiological cells and general complete growth and symmetry and reciprocal relationship with all the other cells of the economy in which it belongs in accordance with the evident design of its creation, this perverted energy begins to create abnormal cells with all the attendant phenomena, which grouped together, we call by the significant title tuberculosis.

It is, therefore, idle to expect to find anything visible which may indicate this tendency to tuberculosis. It is no more rational to expect any such visible manifestation than it is to expect to find on the moving billiard ball before referred to, the manifestation of the secondary impulse which resides in its mass, in advance of the time when it is to produce its own peculiar action upon the ball.

If we examine a fragment of so-called "dead matter," say a crystal of quartz, we find in it a number of properties that must always fill us with surprise and admiration.

In the first place, we find that if the crystal be unsupported it will fall to the ground—that is, it is attracted to the earth—and we call this influence the attraction of gravitation. Here is a perfectly obvious phenomenon, and yet the influence that brings it about is perfectly invisible. No microscope or other instrument will discover its presence in advance of its support being removed from it. Only the balance will make it apparent, and that is only another method of withdrawing from it its support, and is therefore only a result. Here, then, is a force residing within the crystal and perfectly invisible.

Examining the crystal again, we find it to be a hard mass, its particles strongly cohering together. This shows the presence of a second powerful influence, the attraction of cohesion, residing within the crystal and perfectly invisible. But we find this crystal composed of a number of widely different substances. There is silicon, which is a solid; and oxygen, which is a gas; and water, which is a liquid; and water in its turn is composed of two gases, oxygen and hydrogen. The crystal then is made up of the three states of matter—solid, liquid, and gaseous—and this is due to still another and a third form of attraction, chemical affinity residing within the crystal. Yet it is invisible and imperceptible except in its results.

Again looking at the crystal we find that it has a definite shape. It is a six-sided prism, terminated at each end by oblique six-sided pyramids. All uniaxial quartz crystals have this same shape. What is the cause? It lies in the action of a peculiar kind of attraction, the attraction of crystallization or atomic polarity, by virtue of which the atoms arrange themselves in a certain order and direction and develop a peculiar and unvarying shape. But this power or attraction of crystallization, though residing in the crystal, is not visible except in its results, and is not to be determined in advance of its action in any given case.

We observe, too, that the crystal is transparent, insomuch that fine specimens of quartz are ground down to make spectacle lenses, and the Chinese show their skill by grinding large masses of it into spheres of surprising diameter, extraordinary transparency, and great value. What is the source of this transparency? It is the combination of the silica with water under the influence of both the chemical and the crystallographic attractions.

Hang up a crystal of quartz by an unspun silk fibre so that its long axis is horizontal, and directly between the poles of a powerful magnet the crystal will set itself at right angles to a line connecting the poles, and this we call diamagnetism. Here is a mysterious property residing within the crystal that is not visible except by its effects.

Rub the crystal upon a piece of woolen cloth or cat's fur, and it will develop electricity.

Hold it in the sun and then immediately afterward take it into a dark room and it will glow with a faint prosphorescent light.

Select a variety that is called "bi-axial" and look through it at a flame, an open window, or a spot on a piece of white paper, and all these objects, so observed, will appear double and we call it "double refraction." This is one of the phenomena of polarized light.

Trail the crystal over the skin of some nervous woman along the course of some superficial nerve, and she will complain of a slight irritant effect. Set up the crystal in a darkened room and let it be seen by an epileptic or cataleptic patient and she will complain of its burning with a blue flame.

Now all this wonderful assemblage of qualities lies unnoticed within the confines of a crystal, a piece of rock, a fragment of the earth, a morsel of so-called "dead matter." But they are all energies of powerful character. This scrap of the rocky crust of the earth lies in the ground close to the root of a growing stalk of wheat. The wheat seizes upon it, disintegrates it, absorbs, it, builds it into its own structure, and we see it forming a portion of its long, tubular stalk, that surmounted by its beautiful, but heavy head of grains, waves gracefully in the passing breezes.

Turning our attention now to this ear of wheat, we see in its growth the action of a number of forces all imponderable, mysterious, and perfectly independent of man's power. There is the faculty of development by which the stalk forms leaves, flowers, and fruits. There is the chemical energy which generates chlorophyll, starch, gluten, and earthy salts. All these forms of energy are as mysterious as the forces we find in the crystal, only more so.

The grain of wheat on its way to the mill to be ground into flour has in its small mass the promise and potency of a whole stalk of wheat with fifteen or twenty grains. Plant these grains and they will grow twenty times as many more; plant these and a greater number will be produced, and so we may go on until we have raised a whole ten-acre field of wheat from one grain. Thus we have the promise and potentiality of a ship's cargo of wheat from a single grain. This is evident to any one who will take the trouble to think of it, yet because it is an every-day observation, we never trouble our minds with the subject.

Now, if we adopt the mode of thought of Professor Virchow in his assertions concerning tuberculosis, we must deny the truth of the foregoing train of reasoning, because we can find no trace of the future field of wheat in the single parent grain. This is evidently an absurdity. If we pass from the vegetable kingdom to the animal, we find even more complicated energies carrying on processes of growth and development. These energies are perfectly invisible and unrecognizable in advance of the period assigned to them to perform their task, just as the second impulse to the billiard ball does not appear until it is time for it to perform its allotted work.

Recall for a minute all the wonderful phenomena that go on in an animal body. Think of the growth, assimilation, respiration, heart beat, circulation, and various other phenomena. Think of the variety of cell structures: muscular, nervous, and connective tissues; think of the intricate formations; their vast variations; the certainty and accuracy of their construction; think of the mental phenomena and processes of thought, and then recall that all these lie latent and potential in a microscopic, impregnated egg! The mind is staggered by the thought! Yet these phenomena give no sign of their future action in the seemingly simple process of fission, multiplication, and proliferation of the yolk of an egg seen under a high power of the microscope.

If, then, such stupendous potentialities lie hidden in an egg, how can so great a man as Virchow, familiar as he undoubtedly is with all the foregoing considerations, deny the potentiality of tuberculosis in the fœtus because its beginnings are not apparent to his vision armed with the microscope!

Incessant repetition dulls perception to the point of insensibility: familiarity brings about indifference; and so the wonderful potentialities here depicted as incubating in an egg make no impression because so universally and so incessantly observed.

This insensibility has clouded the scientific vision of the great cellular pathologist, and thus he denies a truth which would otherwise inspire him if only brought to him by analogy of the wonderful facts ever before him.

WALTER M. JAMES.

